# Comparison of intraspinal and intrathecal implantation of induced pluripotent stem cell-derived neural precursors for the treatment of spinal cord injury in rats

**DOI:** 10.1186/s13287-015-0255-2

**Published:** 2015-12-22

**Authors:** Takashi Amemori, Jiri Ruzicka, Nataliya Romanyuk, Meena Jhanwar-Uniyal, Eva Sykova, Pavla Jendelova

**Affiliations:** Department of Neuroscience, Institute of Experimental Medicine, Academy of Sciences of the Czech Republic, Videnska 1083, 142 20 Prague 4, Czech Republic; Department of Neuroscience, 2nd Faculty of Medicine, Charles University, Plzenska 130/221, 150 00 Prague 5, Czech Republic; Department of Neurosurgery, New York Medical College, Valhalla, NY 10595 USA

**Keywords:** Spinal cord injury, Human induced pluripotent stem cells, Cell therapy, Cell application route, Neural progenitors, Intraspinal injection, Intrathecal injection

## Abstract

**Background:**

Stem cell treatment provides a promising therapy for patients with spinal cord injury (SCI). However, the applied stem cells exert their effects in different manners that are dependent on the route used for administration.

**Methods:**

In the present study, we administered neural precursors derived from induced pluripotent stem cells (iPS-NPs) either intraspinally into the lesion center or intrathecally into the subarachnoid space of rats with a balloon-induced spinal cord compression lesion. Functional locomotor performance, cell survival, astrogliosis, axonal sprouting and the expression of endogenous neurotrophic growth factors were evaluated using behavioral tests (BBB, flat beam test, rotarod, plantar test), morphometric analysis, immunohistochemistry and qPCR.

**Results:**

Both treatments facilitated the functional locomotor recovery of rats with SCI. iPS-NPs injected intraspinally survived well for 2 months and were positive for MAP2, while cells grafted intrathecally were undetectable at the site of administration or in the spinal cord tissue. Intraspinal implantation increased gray and white matter sparing and axonal sprouting and reduced astrogliosis, while intrathecal application resulted only in an improvement of white matter sparing and an increase in axonal sprouting, in parallel with no positive effect on the expression of endogenous neurotrophic growth factor genes or glial scar reduction.

**Conclusions:**

Intrathecally grafted iPS-NPs had a moderate therapeutic benefit on SCI through a paracrine mechanism that does not require the cells to be present in the tissue; however, the extended survival of i.s. grafted cells in the spinal cord may promote long-term spinal cord tissue regeneration.

**Electronic supplementary material:**

The online version of this article (doi:10.1186/s13287-015-0255-2) contains supplementary material, which is available to authorized users.

## Background

The National Spinal Cord Injury Statistical Center has reported that approximately 66 % of human spinal cord injury (SCI) cases have incomplete lesions caused by vehicular accidents, falls, violence, sports, etc. (https://www.nscisc.uab.edu/reports.aspx). Successful treatment of SCI is difficult owing to the limited regeneration of nervous tissue and its inability to replace lost neurons and injured axons. Different types of stem cells, including embryonic, fetal, and adult stem cells, have been transplanted into animal models of SCI. Cell grafting as a therapeutic strategy for the treatment of SCI is promising, since it can target cell replacement, neuroprotection, and regeneration.

In particular, the use of induced pluripotent stem cells (iPS) is in the center of attention, since these cells can be tailored to patients for autologous use to avoid immune rejection, ethical constraints, and tissue donation [1–4]. Several studies have evaluated the efficacy of iPS-derived neural precursor cells (iPS-NPs) in animal models of SCI (recently reviewed in [3, 5]). When injected into SCI, these cells differentiated predominantly into glia or neurons, formed synapses with host axons and increased regeneration, leading to functional improvement [6–10]. Moreover, specific cell types, such as oligodendrocyte precursors, can be obtained from iPS, which after transplantation can remyelinate host axons following SCI [11, 12]. However, iPS-NPs can also reduce secondary damage through immunomodulation and neurotrophic effects. Human iPS-NPs can spare tissue in the lesion area [7–9], produce cytokines and neurotrophic factors such as neurotrophin 4 (NT4), glia-derived neurotrophic factor (GDNF), and interleukin (IL)-10 [13], and stimulate angiogenesis via the production of vascular endothelial growth factor (VEGF) [7].

In all studies on SCI so far reported, iPS were implanted directly into the spinal cord tissue. However, the direct injection of stem cells into the spinal cord may cause additional damage resulting in further deterioration of the lesioned tissue. In addition, the risk of tumor or teratoma formation is still not negligible, since nestin-positive tumor formation was observed 103 days after the transplantation of iPS-derived neurospheres into a mouse model of SCI. Similarly, teratomas were found after the grafting of murine iPS-derived neurospheres into contusive SCI [14]. On the other hand, the intrathecal (i.t.) application of cells can overcome some of these limitations. Moreover, several studies have shown that intrathecally administered stem cells can partially home into the tissue parenchyma [15–17] while exerting positive therapeutic effects on SCI, even without their long-term survival [18].

Our previous studies have shown that the direct injection of mesenchymal stem cells (MSCs) and/or olfactory ensheathing glia cells as well as adipose-derived MSCs [19, 20], human fetal neural stem cells derived from the spinal cord [21], and human iPS-NPs [9] into a balloon-induced spinal cord compression lesion promotes locomotor recovery. Among all of the cell types used in those previous studies, the application of iPS-NPs resulted in the fastest and most pronounced locomotor recovery accompanied by a high degree of tissue regeneration in a rat model of SCI [9]. iPS-NPs also had a beneficial effect in a rat stroke model [22]. In both studies with iPS-NPs [9, 22], the effect on functional recovery was rapid and preceded grafted cell differentiation and maturation. We therefore assume that a paracrine effect plays an important role in spinal cord regeneration. In the present study, we injected iPS-NPs intrathecally 1 week after SCI, when the hostile environment created by the inflammatory response following SCI is diminished [23, 24]. An intraspinal (i.s.) injection of iPS-NPs into the lesion center at the same time point was used for comparison as a positive control. Behavioral outcome, morphological changes, the expression of neurotrophic factor genes, and axonal regeneration were examined 8 weeks after transplantation (9 weeks after SCI). As a model of SCI, we chose a balloon-induced spinal cord compression lesion, which simulates the pathology caused by the compression of the human spinal cord by an unreduced dislocation or a fracture dislocation of the spine [25]. It is assumed that both mechanical and vascular factors are involved in the pathogenesis of SCI in this model. The balloon compression model is simple and reproducible, and unlike other models (clip injury, weight drop injury) does not require a laminectomy.

## Methods

### Cell preparation

Clone selection, validation of the iPS line, and the derivation of neuronal precursors have been described in detail by Polentes et al. [22]. Briefly, the human iPS line was derived from female (IMR90) human fetal lung fibroblasts (ATCC, Teddington, UK) and transduced with a lentivirus-mediated combination of OCT4, SOX2, NANOG, and LIN28 human cDNA [22]. Early neural precursors were produced in low-attachment culture in the presence of Noggin (500 ng/ml; R&D Systems, Minneapolis, MN, USA), the transforming growth factor-beta pathway inhibitor SB 431542 (10 nM; Sigma, St. Louis, MO, USA), and basic fibroblast growth factor (bFGF; 10 μg/ml) and brain-derived neurotrophic factor (BDNF; 20 μg/ml) (both Pepro Tech, London, UK). Human iPS-NPs were routinely cultured in tissue culture flasks coated with poly-l-ornithine (0.002 % in distilled water) and laminin (10 μg/ml in Dulbecco’s modified Eagle’s medium (DMEM):F12; Sigma). Growth media were comprised of DMEM:F12 and Neurobasal medium (1:1), B27 supplement (1:50), N2 supplement (1:100) (GIBCO, Life Technologies, Grand Island, NY, USA), l-glutamine (2 mM; Sigma), penicillin and streptomycin (50 U/ml; GIBCO), and fibroblast growth factor (FGF; 10 ng/ml), epidermal growth factor (EGF; 10 ng/ml) and BDNF (20 ng/ml) (PeproTech). Prior to transplantation the iPS-NPs were predifferentiated over 7 days in the same medium except for the omission of FGF and EGF [9]. The neural precursors were characterized prior to transplantation. They were negative for the pluripotent markers nanog, SSEA4, and TRA-1-60, slightly positive for oct3/4 (30 %) and sox2 (50 %), and positive (>80 %) for SSEA1, CD133, CD24, CD29, NCAM, nestin, and A2B5. Their full characterization, including mRNA expression, is described by Polentes et al. and Romanyuk et al. [9, 22].

### Animals

Adult male Wistar rats aged 10 weeks and weighing 270–300 g (*n* = 38) were obtained from our facility in the Academy of Sciences of the Czech Republic. All experiments were performed in accordance with the European Communities Council Directive of 22^nd^ of September 2010 (2010/63/EU) regarding the use of animals in research and were approved by the Ethics Committee of the Institute of Experimental Medicine, Academy of Sciences of the Czech Republic.

### Surgical procedure

The animals were anesthetized with isoflurane (Forane; Abbott Laboratories, Queenborough, UK), and the spinal cord was exposed at thoracic vertebra 10. A sterile 2-french Fogarty catheter was inserted into the epidural space until the center of the balloon rested on thoracic vertebra 8 (T8). The balloon was rapidly inflated with 15 μl saline for 5 minutes. During this procedure, 3 % isoflurane in air was administered at a flow rate of 0.3 l/minute, and the animal’s body temperature was kept at 37 °C with a heating pad. After the catheter was deflated and removed, the incised skin and muscle were sutured. The operated animals were assisted in feeding and urination until they had recovered sufficiently to perform these functions on their own. The animals received gentamicin sulfate (5 mg/kg) for 3 days to prevent postoperative infections and were allowed to feed and drink ad libitum.

### Transplantation

One week after the spinal cord was lesioned at T8 by balloon compression, 5 × 10^5^ human iPS-NPs (12^th^ passage) in 50 μl saline (*n* = 9) or 50 μl saline without cells (*n* = 9) were injected intrathecally between L3 and L4 or L4 and L5 through a 25 G needle for 30 seconds. Any remaining cells left in the needle were flushed by an additional injection of 50 μl saline, and the injection needle was left in place for 30 seconds to prevent backflow of the injected content. The position of the needle tip was confirmed by the backflow of cerebrospinal fluid (CSF) into the hub. The i.s. injection of 5 × 10^5^ human iPS-NPs (12^th^ passage) in 5 μl saline (*n* = 9) or 5 μl saline without cells (*n* = 11) was also performed 1 week after SCI. The spinal cord was exposed at T8, and the injection was made in the midline of the spinal cord at a depth of 1 mm below the dorsal surface with an injection rate of 1 μl/minute using a Nano-Injector (Stoelting CO., Wood Dale, IL, USA). The needle was kept in place for a further 5 minutes to prevent leakage of the cell suspension. Cyclosporine A (10 mg/kg), azathioprine sodium (2 mg/kg), methylprednisolone (2 mg/kg, tapered to 0.5 mg/kg), and ampicillin (50–100 mg/kg) were administered 1 day before transplantation and every day throughout the experiments.

### Functional analysis

Animals with and without cell treatment were trained and examined using the plantar, rotarod, and flat beam walking tests before and after surgery during a 2-month observation period. Simultaneously, the animals’ locomotion was evaluated by the Basso, Beattie, and Bresnahan (BBB) scale [26] every week following SCI. The details of these behavioral tests have been published elsewhere [9, 21] but are briefly explained in the following.

The BBB test evaluates joint movement, paw placement, weight support, forelimb–hindlimb coordination, and other parameters using a 0–21 point scale. The plantar test examines the sensitivity of the paw to a noxious thermal stimuli. The withdrawal latency (i.e., how quickly the animal responded to the stimulus) was measured using a Plantar Test apparatus (Ugo Basile, Comerio, Italy). Five repeated measurements were obtained from each hind paw at 5-minute intervals. In the flat beam walking test, the animal was placed at one end of a 1-m-long beam with a flat surface and oriented to walk toward the opposite end of the beam where an escape box was placed. The latency and the trajectory to traverse the beam were recorded by a video-tracking system (TSE-System Inc., Bad Homburg, Germany) for a maximum of 60 seconds. The animals were tested twice a day for three consecutive days. The locomotor performance was evaluated using a 0–7 point scale modified from Metz and Whishaw [27], which enabled the examiner to determine how the animal could walk on the beam (e.g., by dragging their hind legs, using one or two hind legs, etc.). The rotarod test apparatus (Ugo Basile) requires a degree of motor coordination on the part of the tested animals to successfully perform the task. Each animal was placed on a rotating rod at a speed of 10 rpm before surgery and 5 rpm after surgery and was left to walk on the rod for 60 seconds. The animal was given four trials per day with an inter-trial interval of 5 minutes across five consecutive days. The latency to fall off the rod onto a floor plate was measured.

Data are expressed as mean ± standard error of the mean. To compare the data between two groups, a two-sample *t* test for independent samples was performed if the two samples had equal variances. If the samples had unequal variances, the Mann–Whitney test was used for evaluation. The comparison of four groups – i.t. injection of iPS-NPs, i.s. injection of iPS-NPs, and the appropriate saline controls (i.t. and i.s.) – was performed using a two-way analysis of variance. *p* <0.05 was considered statistically significant.

### Histological and immunohistochemical analyses

After completing the behavioral analyses (8 weeks after treatment, 9 weeks after SCI), the animals were deeply anesthetized with ketamine (100 mg/kg) and xylazine (20 mg/kg). Transcardial perfusion was performed with a phosphate buffer solution, followed by a 4 % paraformaldehyde solution in phosphate buffer. A 2-cm-long segment of the spinal cord was dissected between 1 cm cranial and 1 cm caudal to the injury epicenter. Serial cross-sections were obtained with a 5 μm thickness. For morphometric measurements (five i.t.-treated rats, five i.t. control rats, five i.s.-treated rats, seven i.s. control rats), six sections were selected at 1 mm intervals along the craniocaudal axis and stained with Luxol-Fast Blue and Cresyl Violet. Images of each cross-section were taken with an Axioskop 2 plus microscope (Carl Zeiss AG, Oberkochen, Germany) and analyzed by ImageJ software (National Institutes of Health, Bethesda, MD, USA). A series of serial sections were partly stained by antibodies against growth-associated protein (GAP43; Millipore, Billerica, MA, USA) and glial fibrillary acidic protein (GFAP; Sigma). GAP43-positive axons were manually counted. To visualize the reactivity of the anti-GFAP primary antibody, goat anti-mouse IgG conjugated with Alexa-Fluor 594 (Molecular Probes, Eugene, OR, USA) was used. Confocal images were taken with a Zeiss LSM 5 Duo confocal microscope (Car Zeiss AG). To identify human stem cells, antibodies directed against human nuclei (HuNu; Chemicon, Temecula, CA, USA) and human mitochondria (Mitochondrially Encoded Cytochrome C Oxidase II: MTCO2; Abcam, Cambridge, UK) were used. In parallel, the neuronal differentiation of the transplanted cells was examined by microtubule-associated protein 2 (MAP2; Abcam). To visualize primary antibody reactivity, goat anti-mouse IgG conjugated with Alexa-Fluor 488 or 594 (Molecular Probes) was used. Images were taken with an Axioskop 2 plus microscope (Carl Zeiss AG). The remaining group of cell-treated (i.t. *n* = 4, i.s. *n* = 4) and control (i.t. *n* = 4, i.s. *n* = 4) rats were used for PCR analysis. The expression of rat target genes, including brain-derived neurotrophic factor (*Bdnf*), vascular endothelial growth factor A (*Vegfa*), nerve growth factor (*Ngf*), and neurotrophin 3 (*Nt3/Sort1*), were determined by quantitative real-time reverse transcription qPCR in a 7500 Real Time PCR System (Applied Biosystems, Foster City, CA, USA) using TaQMan Gene Expression Master Mix (catalog No4392938) and TagMan Gene Expression Assays 4331182 (Rn02531967_s1/Bdnf/, Rn01511601_ml/Vegfa/, Rn01533872_ml/Ngf/, Rn01521847_ml/Sort1/). The qPCR was carried out in a final volume of 20 μl containing 500 ng extracted RNA. The following thermal profile was used: a single cycle of reverse transcription for 30 minutes at 50 °C and 15 minutes at 95 °C for reverse transcriptase inactivation and DNA polymerase activation, followed by 40 cycles of denaturation at 95 °C for 15 seconds and annealing and extension at 60 °C for 1 minute. The results were analyzed using the integrated 7500 System SDS Software (version 1.3.1; Life Technologies, Carlsbad, CA, USA). Each dataset was normalized with an appropriate TaqMan Endogenous control selected by NormFinder (MOMA, Aarhus, Denmark). As housekeeping genes, Actb (Rn00667869_m1) was chosen for rat target genes. All qPCR reagents were provided by Applied Biosystems. Finally, the data were recalculated to relative quantities and transformed to a log_2_ scale using the Relative Expression Software Tool (REST analysis software; Qiagen, Hilden, Germany) [28]. All numerical data are presented as mean ± standard mean error, and the relative expression ratio of the target genes was compared and analyzed statistically using REST.

## Results

### Functional analysis

#### BBB test

**Fig. 1:**
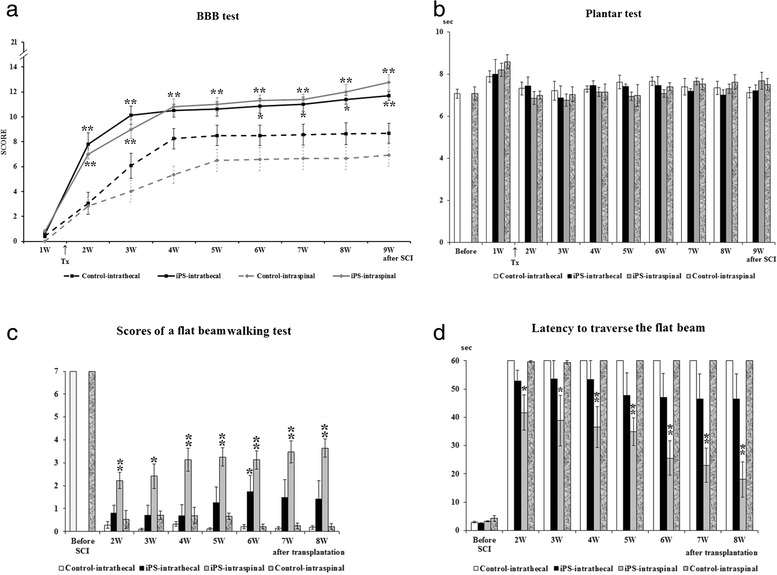
Functional motor recovery evaluated by the BBB test **a**. Statistical differences between the appropriate control and cell-treated groups are indicated by *p <0.05 and **p <0.01. The sensitivity of the hind paws to thermal stimuli was examined by the Plantar test **b**. The withdrawal latency before surgery is represented by the two left-most columns in the figure. There were no significant differences between the controls and the iPS-NP-treated groups throughout the experiment. The flat beam scores evaluate the locomotor function of the hind legs **c**. The maximum score is 7 in a healthy animal. The differences between the i.s. control and cell-treated groups were always significant (**p <0.01 above the gray columns). Locomotor ability to traverse a beam with a flat surface **d**. The latency decreased week by week in the treated groups when compared with the control groups. Statistical differences between the appropriate control and cell-treated groups are indicated: *p <0.05 and **p <0.01. *BBB* Basso, Beattie, and Bresnahan, *iPS* induced pluripotent stem cell, *SCI* spinal cord injury, *W* weeks, *Tx* day of transplantation

The locomotor recovery of the hind legs after balloon compression injury was evaluated weekly using the BBB test. One week after SCI, the rats displayed one of the following: no joint movement, one or two slight joint movements, or maximally extensive one joint movement only, which corresponded to a BBB score of 0–2. The BBB score was calculated from the scores of both hind legs and the BBB score was around 0.5 on average 1 week after surgery. At 1 and 2 weeks following i.t. administration (2 and 3 weeks after SCI), the iPS-NP-injected rats (*n* = 9) showed a rapid and significant improvement of locomotor function in their hind legs (7.8 ± 0.9 and 10.1 ± 0.7, respectively) compared with the saline-injected controls (*n* = 9) (3.1 ± 0.9 and 6.0 ± 1.0, respectively) (*p* <0.01) (Fig. 1a). However, further improvement in the BBB score of the i.t. cell-injected animals was not so pronounced in subsequent weeks and correlated with their functional recovery, which did not advance as rapidly as it did during the first 2 weeks after treatment. A statistically significant difference in the BBB scores of the cell-injected and saline-injected animals was seen again from week 5 to the end of the experiment (*p* <0.05). The i.s. iPS-NP-treated group (*n* = 11), similarly to the i.t.-treated one, showed a significant improvement 1 week after treatment (1.1 ± 0.4) (*p* <0.01) followed by a gradual continuous improvement between 3 and 7 weeks after SCI (*p* <0.05), with the greatest improvement observed during the 8th and 9th weeks after SCI (*p* <0.01). The final scores were 8.7 ± 0.8 and 6.9 ± 0.9 in the i.t. (*n* = 9) and i.s. (*n* = 9) control groups, respectively, and 11.7 ± 0.4 in the i.t. iPS-NP-treated group and 12.8 ± 0.6 in the i.s. iPS-NP-injected group.

#### Plantar test

In this test, the sensitivity of the hind paw to a noxious thermal stimulus was evaluated before SCI and every week after SCI (Fig. 1b). The withdrawal latency was approximately 7.1 seconds before SCI. The latency in responding to the stimulus was slightly prolonged after surgery in the i.t. control and iPS-NP-treated groups. Their withdrawal latencies gradually returned to the intact values, but were very unstable in subsequent measurement. The final observed latencies were 7.1 ± 0.3 and 7.5 ± 0.3 seconds in the i.t. and i.s. control groups, respectively, 7.2 ± 0.3 seconds in the i.t. iPS-NP-treated group, and 6.5 ± 0.2 seconds in the i.s. iPS-NP-treated group. There were no significant differences between the control and cell-treated groups during the 9-week observation period (*p* >0.05). No animals in any group showed any hypersensitivity or allodynia throughout the experiments.

#### Beam walking test

The locomotor performance of the hind legs while traversing the beam was evaluated using a 0–7 point scale modified from Metz and Whishaw [27] before surgery and every week from the second week after treatment. The rats generally tried to traverse the beam during the first trial after the induction of the spinal cord lesion. When they tried to move forwards to reach the escape box, they fell off the beam because of a lack of locomotor function in their hind legs. If they could not improve their locomotor function during subsequent experiments, they appeared to become discouraged by their repeated failures and their fear of falling. They gave up trying to traverse the beam and instead remained at the same point on the beam where they were initially placed. This behavior was clearly seen in all control rats, independent of the route of saline injection. The scores at the beginning of the series of measurements after SCI were therefore better than those observed in the later measurements (Fig. 1c). In contrast, the rats that received cell treatment maintained their efforts to perform the task and displayed gradual functional recovery. A statistically significant difference was detected between the i.t. iPS-NP-treated and control groups (*p* >0.05) in week 6, while in weeks 5, 7, and 8 a strong trend towards improvement was observed. The final scores were 1.4 ± 0.8 out of 7 (the maximum for intact animals) in the treated group and 0.2 ± 0.1 in the control group. The scores in the i.s. iPS-NP-treated animals were always higher than those in the control group throughout the experiments (*p* <0.01).

Simultaneously, the latency to traverse a beam with a flat surface was examined before SCI and every week from the second week after treatment. Before surgery, animals could easily traverse the beam in 3.0 ± 0.2 seconds on average (Fig. 1d). After lesioning, they lost their ability to traverse the beam using their hind legs and mainly stayed at the same point of the beam where they were placed. When they tried to move forwards to reach the escape box that was placed at the opposite end of the beam, they generally fell off the beam. This situation continued throughout the experiments in both groups of control rats. In contrast, the rats which received an i.t. injection of iPS-NPs improved their ability to traverse the beam week by week, but this improvement was not statistically significant (*p* >0.05). Their final latency was 46.4 ± 8.9 seconds at 8 weeks after treatment (9 weeks after SCI). The animals with i.s. treatment started to traverse the beam as early as 2 weeks after treatment, and their latency continuously decreased during the 8 weeks of observation. There were significant differences between the i.s. control and iPS-NP-treated groups between 2 weeks and 3 weeks after treatment (*p* <0.05) and between 4 weeks and 8 weeks after treatment (*p* <0.01). At the end of the series of experiments, two out of eight rats could completely traverse the beam in the i.t. iPS-NP-treated group, while seven out of eight rats could reach the opposite end of the beam in the i.s. iPS-NP-treated group. The final latency was 18.0 ± 6.2 seconds in the i.s. iPS-NP-treated animals. However, when comparing only those rats from both cell-treated groups that were able to accomplish the task, there were no differences in latencies between the i.t. and i.s. cell-treated animals.

#### Rotarod test

Motor coordination was assessed using the rotarod test before surgery at a speed of 10 rpm and every 2 weeks after treatment at a speed of 5 rpm. After surgery, the animals lost their ability to walk on the rotating rod. The time spent on the rod until falling off onto the floor plate was 3.2 ± 0.4 and 3.3 ± 0.5 seconds in the two control groups, 4.6 ± 1.0 seconds in the i.t. iPS-NP-treated group, and 11.7 ± 4.8 seconds in the i.s. iPS-NP-treated group (*n* = 8) at 2 weeks after treatment (3 weeks after SCI). Similar to their performance in the beam test, the animals actively tried to keep their bodies on the rod using their front legs, which prevented them from dropping as they were able to grasp groves in the rod with their nails during the first trials after treatment. As the control rats could not recover locomotor function in their hind legs, their latency gradually decreased in subsequent measurements (Additional file 1: Figure S1). Conversely, the i.t. iPS-NP-treated group increased their time spent on the rod using their gradually recovering hind legs, although no significant differences between the i.t. control and iPS-NP-treated groups were observed throughout the experiment (*p* >0.05). Similar results were obtained for the i.s. iPS-NP-treated animals, although these animals did improve their performance in the rotarod test week by week and performed better than the i.t.-treated animals. However, no statistically significant differences were found between any of the groups (*p* >0.05).

### Cell survival and differentiation

**Fig. 2:**
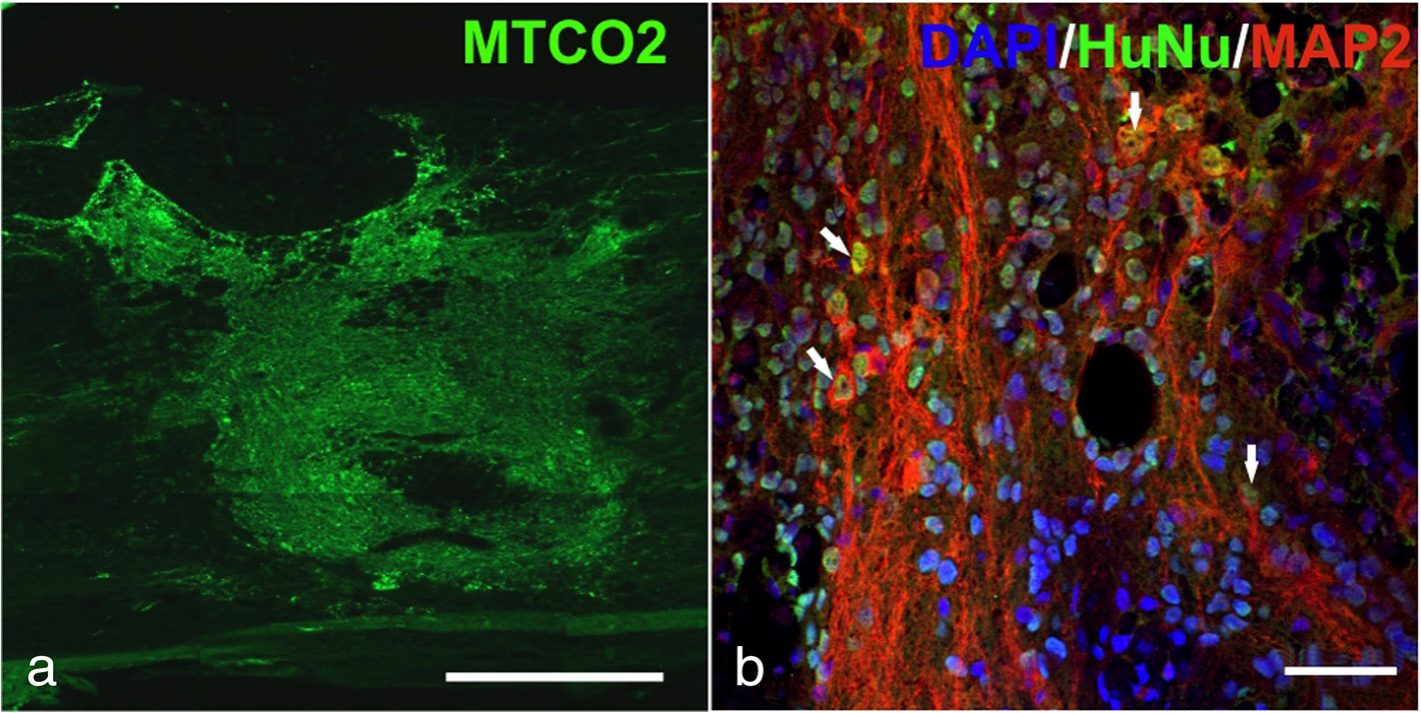
MTCO2-positive transplanted cells in the host spinal cord 8 weeks after i.s. implantation of iPS-NPs. Scale bar = 500 μm **a**. HuNu-positive transplanted cells (*green*) express the neuronal marker MAP2 (*red*). Nuclei are stained by DAPI (*blue*). Colocalization is marked by *arrows*. Scale bar = 50 μm **b**. *DAPI* 4',6-diamidino-2-phenylindole, *HuNu* human nuclei, *MAP2* microtubule-associated protein 2

HuNu-positive or MTCO2-positive grafted iPS-NPs were found at 8 weeks after transplantation in the i.s.-treated rats (Fig. 2). The surviving grafted cells, which were detected by their expression of the human mitochondrial marker MTCO2 and recognized as a mass of bright green fluorescence, accumulated in large number at the injection site with no tumor or tumor-like formation (Fig. 2a). Grafted human iPS-NPs were also detected by the human-specific nuclei protein HuNu, identified as green nuclei and surrounded by red cytoplasm positive for the neuronal marker MAP2 (Fig. 2b). Neither HuNu-positive nor MTCO2-positive cells were identified on the surface of the pia matter or in the host parenchyma of the spinal cord in the intrathecally transplanted animals.

### Morphometric measurements

**Fig. 3:**
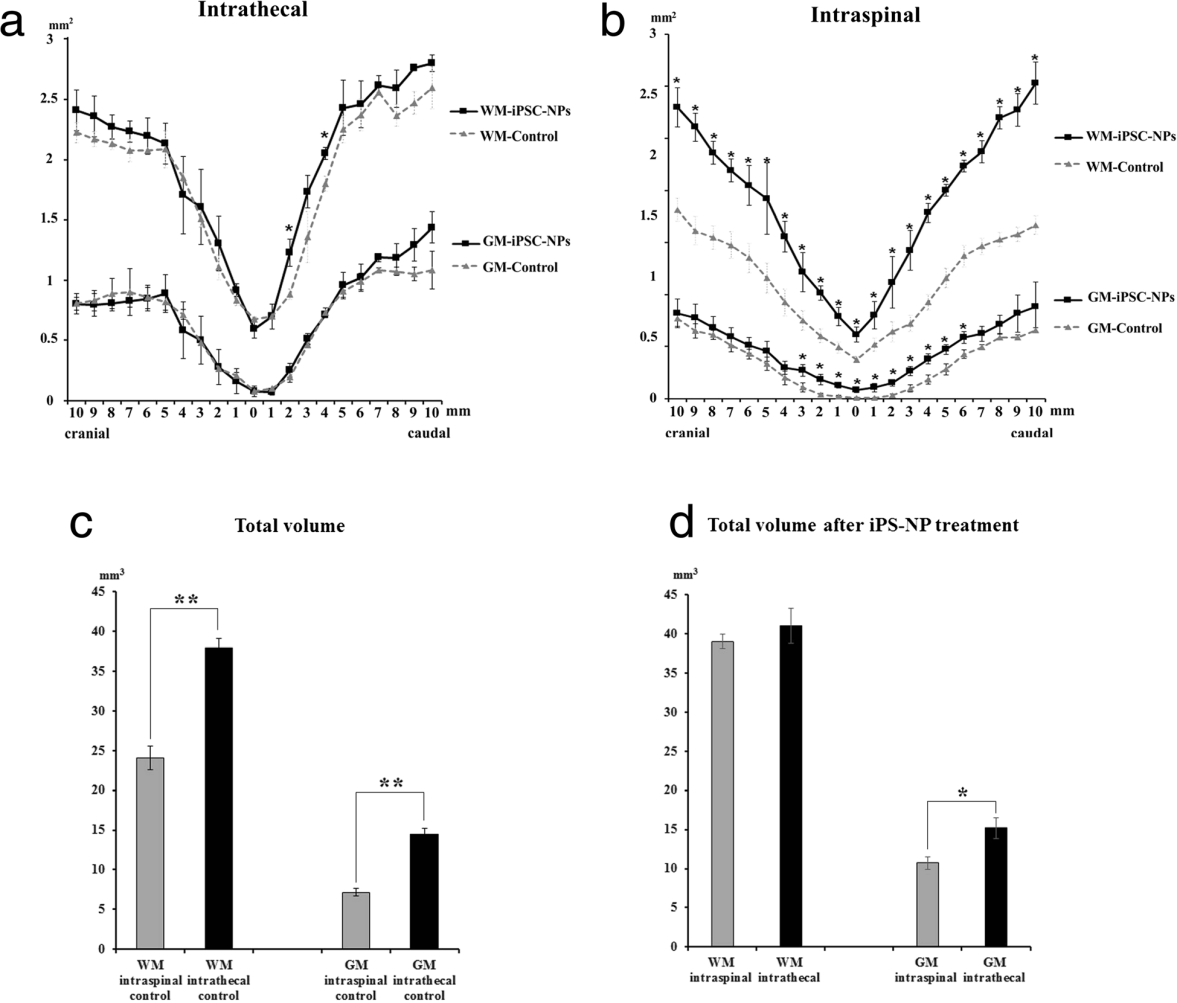
Morphometric measurement of the white matter (*WM*) and gray matter (*GM*). The cross-sectional area (mm^2^) is plotted at 1 mm increments in both the cranial and caudal directions from the injury epicenter, which is indicated by the number 0. The WM was significantly spared only 2 and 4 mm caudal to the injury epicenter in the i.t.-treated group **a**. In contrast, the WM and GM were prominently spared in the i.s. iPS-NP-transplanted group **b**. The total volume of a 2-mm-long spinal segment, including the injury epicenter, is compared between the i.s. and i.t. control groups **c**. Statistically significant differences were found in both the volume of the WM and also the volume of the GM (**p <0.01). Total volume of a 2-mm-long spinal segment after iPS-NP treatment **d**. There is a significant difference in the volume of the GM between i.s. and i.t. administration (*p <0.05). *iPSC-NP* neural precursors derived from induced pluripotent stem cells

Eight weeks after treatment (9 weeks after SCI), the experiments were terminated and the cross-sectional area of spared white and gray matter was measured from a 2-cm-long spinal cord section, excised between 1 cm cranial and 1 cm caudal to the injury epicenter, which was recognized as the smallest area of the spinal cord. The cross-sectional areas (mm^2^) were plotted at 1 mm increments from the injury epicenter, which is indicated by 0 in Fig. 3a, b. When the total volume of the whole length of the extracted spinal cord segment was compared between the i.s. and i.t. control groups, significant differences were apparent in the extent of white and gray matter sparing (*p* <0.01) (Fig. 3c). In the animals that received i.t. injections (control group, *n* = 5; transplanted group, *n* = 5), a significant difference was found in the cross-sectional area of the white matter at 2 and 4 mm caudal to the injury epicenter (*p* <0.05), while there was no statistical difference in the gray matter between the two groups (*p* >0.05) (Fig. 3a). The intraspinally grafted rats (*n* = 5) showed that the white matter was spared more than in the control animals (*n* = 7) throughout the whole length of the 2-mm-long spinal segment, including at the injury epicenter (*p* <0.05), and that the gray matter was significantly spared mainly in the region between 3 mm cranial and 6 mm caudal to the injury epicenter, where the iPS-NPs had been implanted (*p* <0.05) (Fig. 3b). Differences also appeared in the total volume of gray matter after cell treatment, since the i.t. cell-treated spinal cord tissue was spared to a greater extent than the i.s. cell-treated tissue (Fig. 3d).

### In vivo tissue analysis

**Fig. 4:**
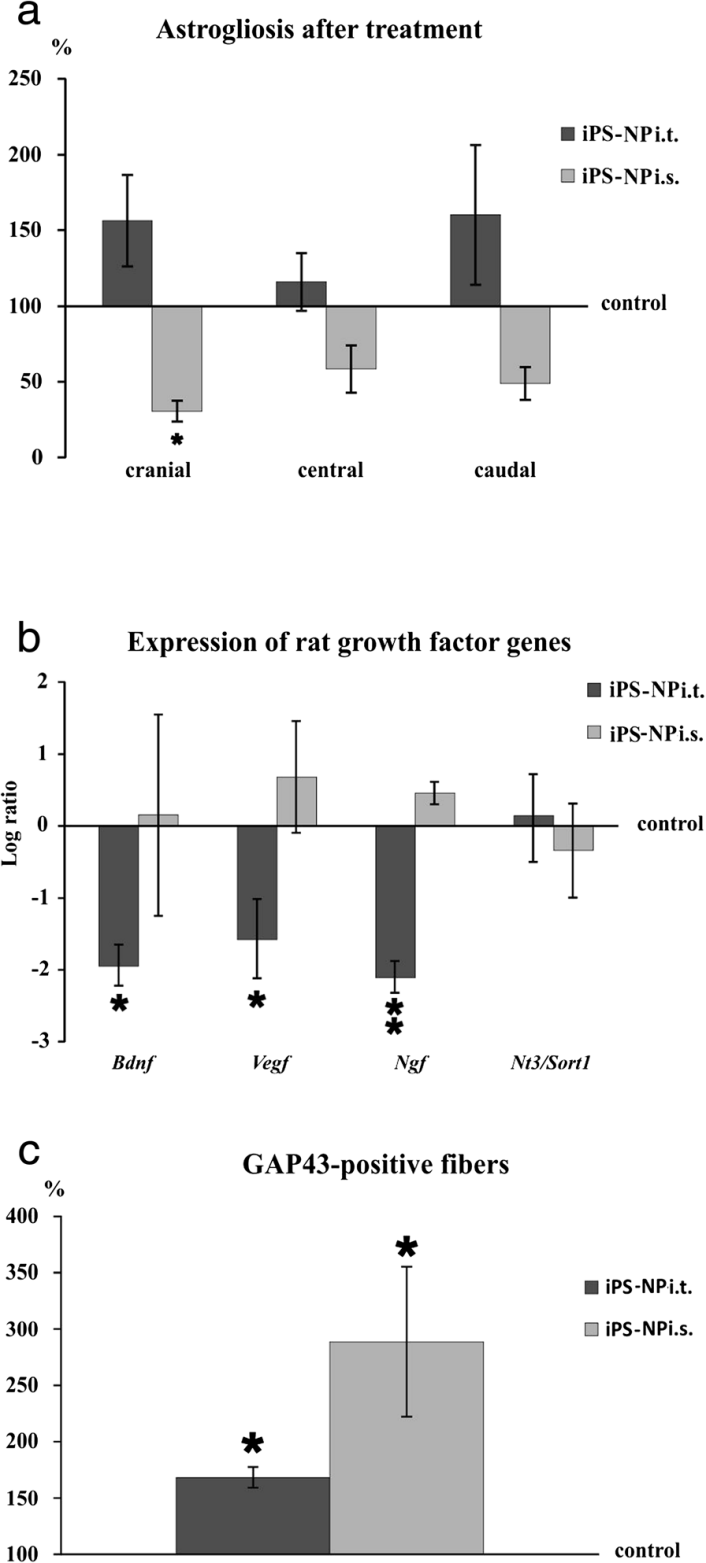
GFAP-positive areas are compared in three different parts of the spinal segment: cranial, between 3 and 7 mm cranial; central, between 2 mm cranial and 2 mm caudal; and caudal, between 3 and 7 mm caudal to the injury epicenter **a**. The average GFAP-positive area around the lesion in the appropriate control group is shown as the baseline of 100 %. Rat gene expression of BDNF, VEGF, nerve growth factor (NGF), and neurotrophin 3 (NT3) analyzed by qPCR **b**. The level of the control expression is indicated by the number 0. An increase or decrease from this level is shown as positive or negative scale numbers, respectively. The levels of BDNF, VEGF, and NGF in the i.t. iPS-NP-treated group are significantly lower than those in the control animals. There was no significant difference between the i.s. iPS-NP-treated and control groups in terms of gene expression. Axonal sprouting evaluated by GAP43-positive fibers **c**. The average number of positive fibers in the control group is shown as the baseline of 100 %. Both cell-treated groups show the increased expression of GAP43, which was more pronounced in the i.s. cell-treated animals. *iPS-NP* neural precursors derived from induced pluripotent stem cells, *i.s.* intraspinal, *i.t.* intrathecal

SCI-induced astrogliosis is an important process in glial scar formation, as reactive astrocytes increase their expression of GFAP. The size of the GFAP-positive area was determined from serial cross-sections at 1 mm increments from the injury epicenter 2 months after i.t. or i.s. treatment. The results were separated into cranial (3–7 mm cranial to the injury epicenter), central (between 2 mm cranial and 2 mm caudal to the injury epicenter), and caudal (3–7 mm caudal to the injury epicenter) areas. The i.t. injection of iPS-NPs did not exert any effects against scar formation. There was no significant difference between the i.t.-injected groups in any of the investigated areas (*p* >0.05) (Fig. 4a). In the i.s. iPS-NP-treated group, on the other hand, astrogliosis was significantly reduced in the cranial area when comparing the i.s.-treated and control groups (*p* <0.05) (Fig. 4a).

To evaluate the neurotrophic effects of the treatment, the gene expression of rat neurotrophic factors, including BDNF, VEGF, nerve growth factor (NGF), and neurotrophin 3 (NT3), was analyzed by qPCR at 8 weeks after treatment (9 weeks after SCI). There was a significant downregulation of BDNF, VEGF, and NGF gene expression (*p* <0.05) and no effect on NT3 expression (*p* >0.05) in the i.t. iPS-NP-treated group compared with the control group (Fig. 4b). In the intraspinally treated animals, the gene expression of BDNF, VEGF, and NGF was higher than that of NT3 and lower than in the control animals, but statistically significant differences were not found between the i.s. iPS-NP-treated and saline-injected group (*p* >0.05).

The elevated expression of GAP43 leads to axonal sprouting in the spinal cord [23]. To assess axonal regeneration and sprouting, the number of GAP43-positive fibers was counted from serial cross-sections. If the number of GAP43-positive fibers in the control group is considered as 100 %, the number of positive fibers increased by 168.3 ± 9.1 % in the i.t. iPS-NP-treated group and 288.7 ± 66.5 % in the i.s. iPS-NP-treated group (Fig. 4c). GAP43-positive fibers were seen more abundantly in the i.t. cell-treated group than in the saline-treated control group, and even more GAP43-positive fibers were present in the i.s. cell-treated animals.

## Discussion

Our study, for the first time, evaluated the i.t. application of iPS-NPs into a rat model of SCI. We have shown that the i.t. injection of iPS-NPs can improve functional locomotor outcome as determined not only by BBB analysis, but also, to some extent, by the flat beam test. This implies that the i.t.-treated animals could recover the ability to support their body weight by using their hind legs, but more sophisticated and complex functions, such as coordination between the forelimbs and hindlimbs, more precise paw placement, and maintaining their balance by using their legs, recovered fully in only 25 % of the i.t. cell-treated animals. On the other hand, the i.s. injection of iPS-NPs improved the functional outcome in all of the locomotor tests. In agreement with our previous findings, intraspinally injected iPS-NPs survived well for the whole observation period [9]. We could not trace any surviving grafted cells using human nuclei and mitochondria markers (HuNu, MTCO2) 8 weeks after i.t. injection. The inability to detect surviving cells may be owing to the fact that these transplanted cells were widely dispersed in the CSF or that the conditions in the CSF were unfavorable for the long-term survival of the transplanted cells. However, cell survival after i.t. application could be dependent on the cell type used and/or on the time window in which the cells were administered. Mothe et al. [29] compared intrathecally transplanted neural stem/progenitor cells (NSPCs) and bone marrow stromal cells (BMSCs) in a clip model of SCI. By four weeks after transplantation, more NSPCs had migrated to the lesion site relative to BMSCs and uninjured animals. However, there was no preferential homing of either of these types of cells into the parenchyma of the injury site, and most of the transplanted cells remained in the i.t. space. By 4 weeks post transplantation, significantly more NSPCs were found at the site of injury (T8), while the majority of BMSCs were found at the injection site (L3–L5). Similarly, when MSCs were transplanted intrathecally at the lesion site by a catheter guided from the cisterna magna 1 week after a balloon compression lesion, the majority of the transplanted cells were seen attached to the pia mater 28 days after SCI, with only a few cells having penetrated into the white matter [30]. We reported previously only a few human MSCs attached to the dorsal surface of the lesioned spinal cord 2 weeks after i.t. application with no survival of cells 8 weeks after application. However, we detected white matter sparing, remodeling of the glial scar, and a decrease in the levels of inflammatory cytokines in the center of the lesion along with enhanced locomotor function [31].

The morphometric measurements revealed that tissue sparing was not as marked in the white and gray matter after i.t. treatment compared with the intraspinally injected animals. Significant tissue sparing was observed in the area of the spinal cord caudal, but not cranial, to the injury epicenter after treatment. This could be explained by the fact that transplanted cells which were injected in the lumbar area may not have been able to reach beyond the balloon-compressed site because the subarachnoid space may have narrowed or collapsed as SCI damage progressed. The direct injection of iPS-NPs is likely to cause tissue damage around the injection site. The volumes of white and gray matter were reduced more in the i.s.-injected controls than in the controls with an i.t. injection, indicating further damage due to the more invasive surgical procedure. Moreover, the volume of the gray matter was spared significantly more in the i.t.-treated group than in the i.s.-treated group. This finding corresponded to lower BBB scores in i.s. controls than in i.t. controls; however, this result was not statistically significant. The beneficial effect of i.s. application might be influenced by the fact that saline-injected i.s. animals scored worse in the BBB test and their spinal cord tissue was more damaged than in i.t.-injected control rats. It is worth mentioning that the tissue cavity which develops after lesioning was reduced or partially filled with grafted cells after i.s. treatment, but i.t.-injected cells were not able to home into the lesion. The volumes of the white and gray matter in i.s. cell-injected animals were analyzed and calculated from a segment of the spinal cord that included transplanted cells, since grafted iPS-NPs incorporated nicely into the host tissue. However, it was impossible to identify transplanted cells in the cross-sections stained with Luxol-Fast Blue and Cresyl Violet, which were used to distinguish the white and gray matter.

Axonal regeneration was facilitated by i.t.-injected cell treatment, but was not accompanied by the upregulation of growth factors in the host tissue of the spinal cord 2 months after grafting (Fig. 4c). Rat neurotrophic factors, including BDNF, VEGF, NGF, and NT3, did not significantly change after i.s. injection of iPS-NPs compared with those in the control animals. However, we observed previously the upregulated expression of human neurotrophin genes for NGF, FGF8, and GDNF [9]. Improved axonal sprouting was therefore most probably supported by exogenous neurotrophic factors rather than host neurotrophins. However, our analysis was performed at the end of the study. It is likely that a transient upregulation of host neurotrophic factors might be observed at earlier time points after cell application.

Direct injection of iPS-NPs into the host tissue has been shown to be effective not only in a model of SCI [9], but also in the middle cerebral artery occlusion model of stroke [22]. In both studies, the grafted cells differentiated into tissue-specific neurons (striatal dopaminergic neurons or interneurons and motoneurons); and in both settings, the injection of iPS-NPs had a dual effect that resulted in rapid functional improvement followed by slow cell differentiation and partial reconstruction of the impaired pathways. Similarly, in the studies by Lu et al. [32, 33] intraspinally transplanted human iPS-derived neural stem cells successfully extended large numbers of axons even over long distances and formed synapses with host neurons after complete spinal transection or hemisection. In our study, it seems that the transplanted cells could not directly reach the lesioned spinal cord via the i.t. route, and therefore this route of administration was not as effective as the i.s. route. However, there was a paracrine effect of iPS-NPs that did not require the presence of cells in the tissue. This paracrine effect was strong enough to improve functional outcome and axonal sprouting and to partially spare the damaged tissue. The obtained results are comparable with the effect of i.t.-injected MSCs and NSPCs [29, 31]. Cells dispersed in the CSF will be less likely to make the contacts necessary for tumor or teratoma formation, which has to be taken into consideration for i.s. application.

## Conclusion

From the results of the present study, it is evident that i.t. application of iPS-NPs had a moderate therapeutic influence (resulting in locomotor improvement, moderate tissue sparing, and axonal sprouting) on SCI through a paracrine mechanism that does not require the presence of cells in the tissue. This effect was comparable with that of other studies reporting similar results after i.t. treatment using MSCs or NSPCs in SCI. Further studies are still required to answer the question of whether repeated i.t. injections and/or an increase in the number of cells transplanted can exert even a more beneficial effect in the treatment of SCI.

## Additional file

Additional file 1: Figure S1. Showing locomotor coordination evaluated by the rotarod test. The time spent on the rod gradually increased in both treated groups, but there were no statistically significant differences between the control and treated groups. (JPG 42 kb)

## Abbreviations

BBB: Basso, Beattie, and Bresnahan
BDNF: Brain-derived neurotrophic factor
bFGF: Basic Fibroblast growth factor
BMSC: Bone marrow stromal cell
CSF: Cerebrospinal fluid
DMEM: Dulbecco’s modified Eagle’s medium
EGF: Epidermal growth factor
FGF: Fibroblast growth factor
GAP: Growth-associated protein
GDNF: Glia-derived neurotrophic factor
GFAP: Glial fibrillary acidic protein
HuNu: Human nuclei
i.s.: Intraspinal
i.t.: Intrathecal
IL: Interleukin
iPS: Induced pluripotent stem cell
iPS-NP: Neural precursor derived from induced pluripotent stem cells
MAP2: Microtubule-associated protein 2
MSC: Mesenchymal stem cell
MTCO2: mitochondrially encoded cytochrome c oxidase II
NGF: Nerve growth factor
NSPC: Neural stem/progenitor cell
NT3: Neurotrophin 3
NT4: Neurotrophin 4
SCI: Spinal cord injury
T8: Thoracic vertebra 8
VEGF: Vascular endothelial growth factor
