# Supplementary figures and images for: Comparison of intraspinal and intrathecal implantation of induced pluripotent stem cell-derived neural precursors for the treatment of spinal cord injury in rats
Source: Stem Cell Res Ther. 2015 Dec 22;6:257. doi: 10.1186/s13287-015-0255-2 (PMC4688936; doi:10.1186/s13287-015-0255-2)

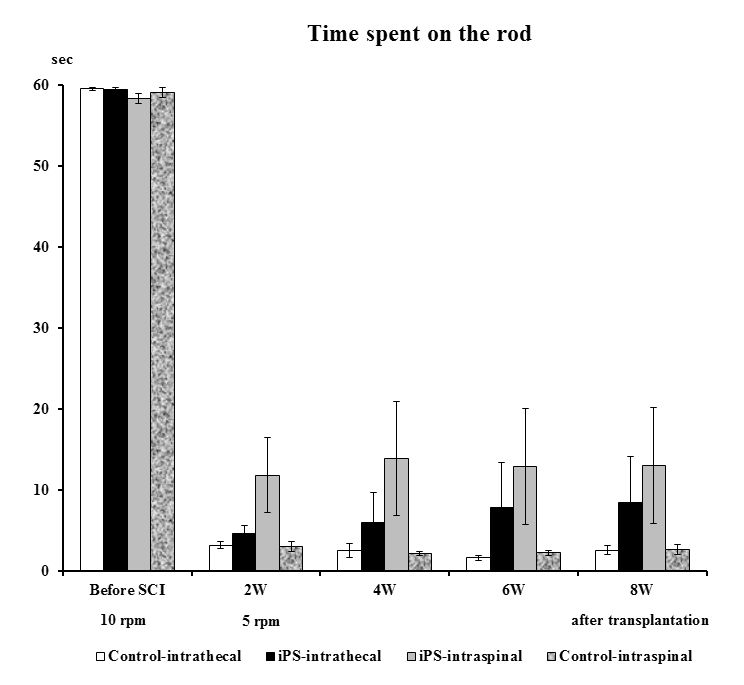

Supplement: Additional file 1: Figure S1. — Showing locomotor coordination evaluated by the rotarod test. The time spent on the rod gradually increased in both treated groups, but there were no statistically significant differences between the control and treated groups. (JPG 42 kb) [file 13287_2015_255_MOESM1_ESM.jpg]
